# Liver cirrhosis in a patient with hepatic hereditary hemorrhagic telangiectasia and Budd–Chiari syndrome: a case report

**DOI:** 10.1186/s12876-020-01311-1

**Published:** 2020-06-03

**Authors:** Bai-Guo Xu, Jing Liang, Ke-Feng Jia, Tao Han

**Affiliations:** 1 Department of Hepatology and Gastroenterology, the Third Central Clinical College of Tianjin Medical Universityssss, 83 Jintang Road, Hedong District, Tianjin, 300170 China; 2 Tianjin Key Laboratory of Extracorporeal Life Support for Critical Diseases; Artificial Cell Engineering Technology Research Center, Tianjin, China; 3 Tianjin Institute of Hepatobiliary Disease, Tianjin, China; 4 Department of Radiology, the Third Central Clinical College of Tianjin Medical University, Tianjin, 300170 China

**Keywords:** Hereditary hemorrhagic telangiectasia, Hepatic hereditary hemorrhagic telangiectasia (HHHT),Budd–Chiari syndrome, Cirrhosis, Case report

## Abstract

**Background:**

Hereditary hemorrhagic telangiectasia (HHT) often involves the liver, and belongs to abnormal blood vessel disease. The etiology of Budd–Chiari syndrome (BCS) is not clear, but congenital vascular dysplasia is considered to be one of the causes. Liver cirrhosis due to hepatic hereditary hemorrhagic telangiectasia concomitant with BCS has not been reported. Here, we report a case of cirrhosis with hepatic hereditary hemorrhagic telangiectasia (HHHT) and BCS.

**Case presentation:**

A 58-year-old woman with hepatic hereditary hemorrhagic telangiectasia showed decompensated liver cirrhosis, and abdominal imaging revealed Budd–Chiari syndrome. Disease has progressed considerably during 2.5 years after hospital discharge despite subsequent transjugular intrahepatic portosystemic shunting (TIPS). One hypothesis that might explain the coexistence of hepatic hereditary hemorrhagic telangiectasia and Budd–Chiari syndrome in this patient is ischemia and thrombosis of hepatic veins.

**Conclusions:**

Further studies are required to evaluate the relationship between HHHT and BCS. Our observations already challenged the TIPS therapeutic strategy in BCS secondary to HHHT patients.

## Background

Hereditary hemorrhagic telangiectasia (HHT, previously Osler–Weber–Rendu syndrome) is an autosomal dominant disease characterized by telangiectasias in the skin and mucous membranes and arteriovenous malformations in the lungs, brain, gastrointestinal tract, and liver. If HHT involves the liver, Patients often have 3 types of intrahepatic shunts: hepatic artery–hepatic vein, hepatic artery–portal vein, and portal vein–hepatic vein, and may be accompanied by cirrhosis and related complications. The etiology of BCS is not clear, but congenital vascular dysplasia is considered to be one of the causes [1]. Both of the disease have the similar etiology. Liver cirrhosis due to hepatic hereditary hemorrhagic telangiectasia (HHHT) concomitant with BCS has not been reported, and the relationship between them is worth further clarification. Here, we report a case of cirrhosis with HHHT and BCS.

## Case presentation

A 58-year-old woman was referred to hospital for abdominal distension of > 1-month duration. With a history anemia for > 10 years, she suffered intermittent bleeding of the nose and tongue which were diagnosed as HHT. Her mother, elder brother, and sister all suffered from HHT. They all had typical clinical symptoms and signs of HHT, such as repeated nasal bleeding and capillaries on face and lips. Her mother died of intracranial hemorrhage. The elder brother died of upper gastrointestinal bleeding with liver cirrhosis and refractory ascites. As far as we known her sister had not exact splanchnic vascular malformation but repeated nasal bleeding.

**Fig. 1:**
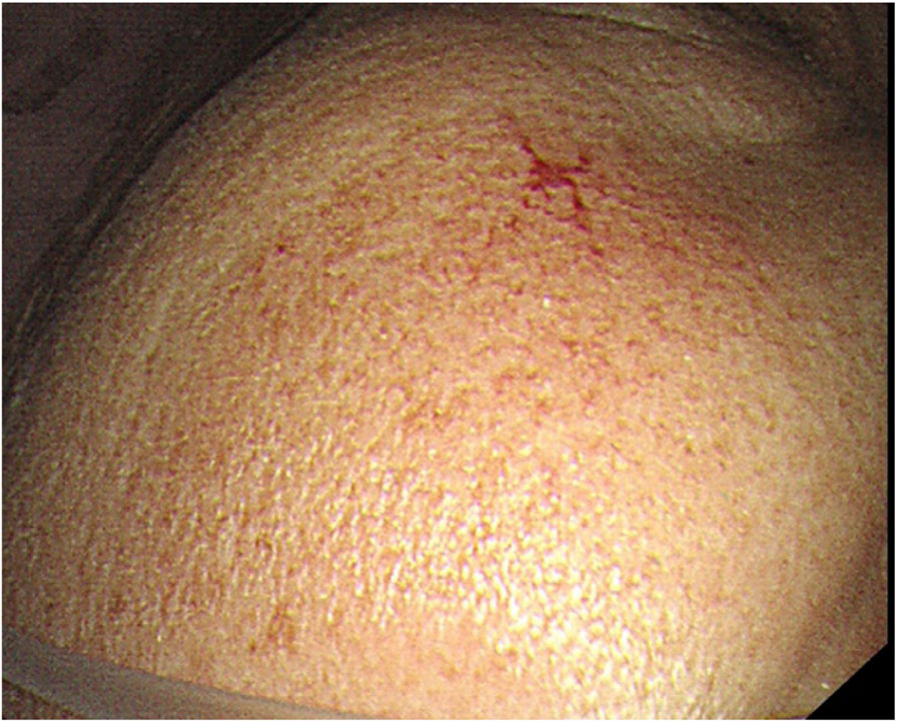
Physical examination of her cheek: Cheek has a reticulated capillary that can fade under pressure

Physical examination showed her cheek (Fig. 1), fingers of both hands and tongue tip to have multiple stripes/reticulated capillaries. Shifting dullness was documented, Edema was negative in both lower limbs. Laboratory examination showed the hemoglobin level to be 86 g/L (normal range: 110–150 g/L). Levels of albumin, alanine aminotransferase, aspartate aminotransferase and bilirubin were in the normal range. The level of alkaline phosphatase was 227 U/L (normal range, 50–135 U/L). Serum levels of copper and iron were normal, as were blood levels of immune- globulin (Ig) G and IgM. Tests for hepatitis-B surface antigen, hepatitis-B e antigen, hepatitis-C antibody and immunologic tests were negative.

**Fig. 2:**
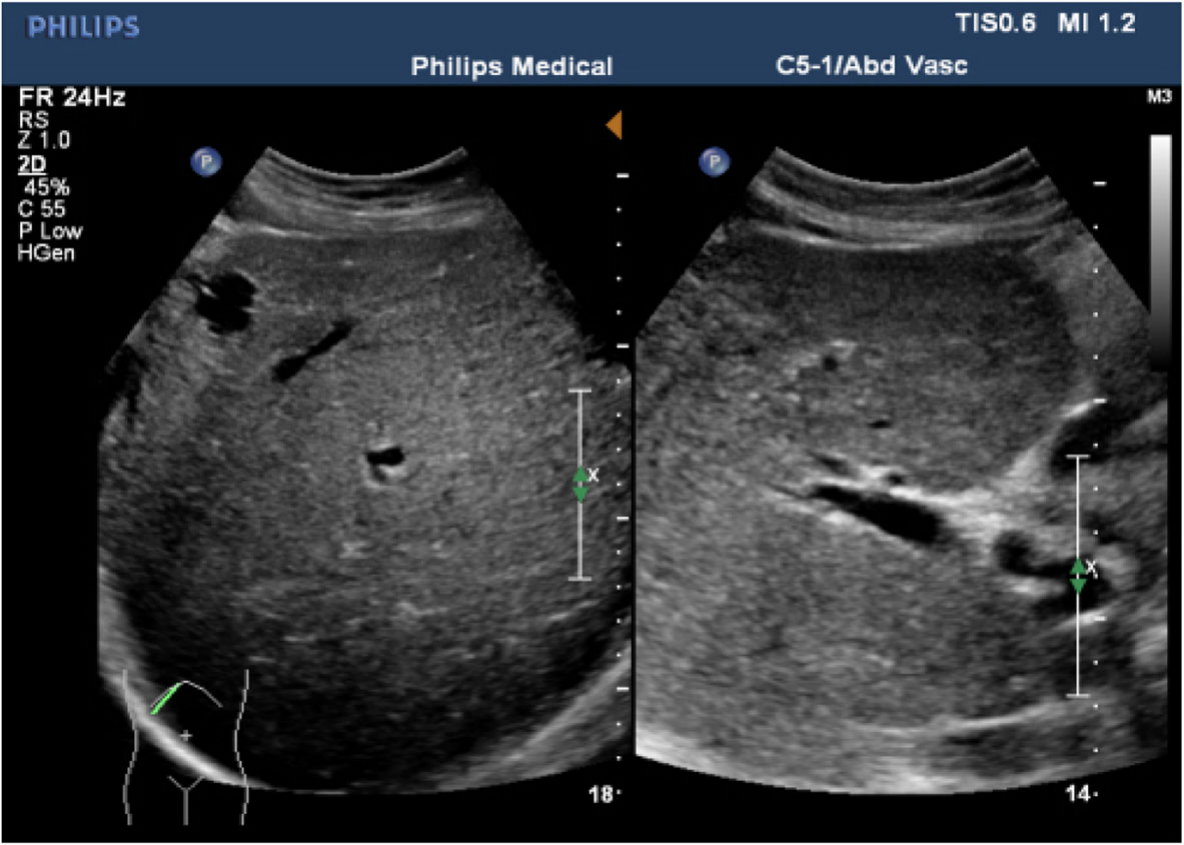
B-ultrasound of the abdomen: Ultrasound shows the left hepatic vein to be narrow (~ 0.5 cm in inner diameter). The right hepatic vein is narrow (the inner diameter is ~ 0.38 cm). The middle hepatic vein is occluded (inner diameter is ~ 0.38 cm; the distal end is dilated, with an inner diameter of ~ 0.8 cm)

**Fig. 3:**
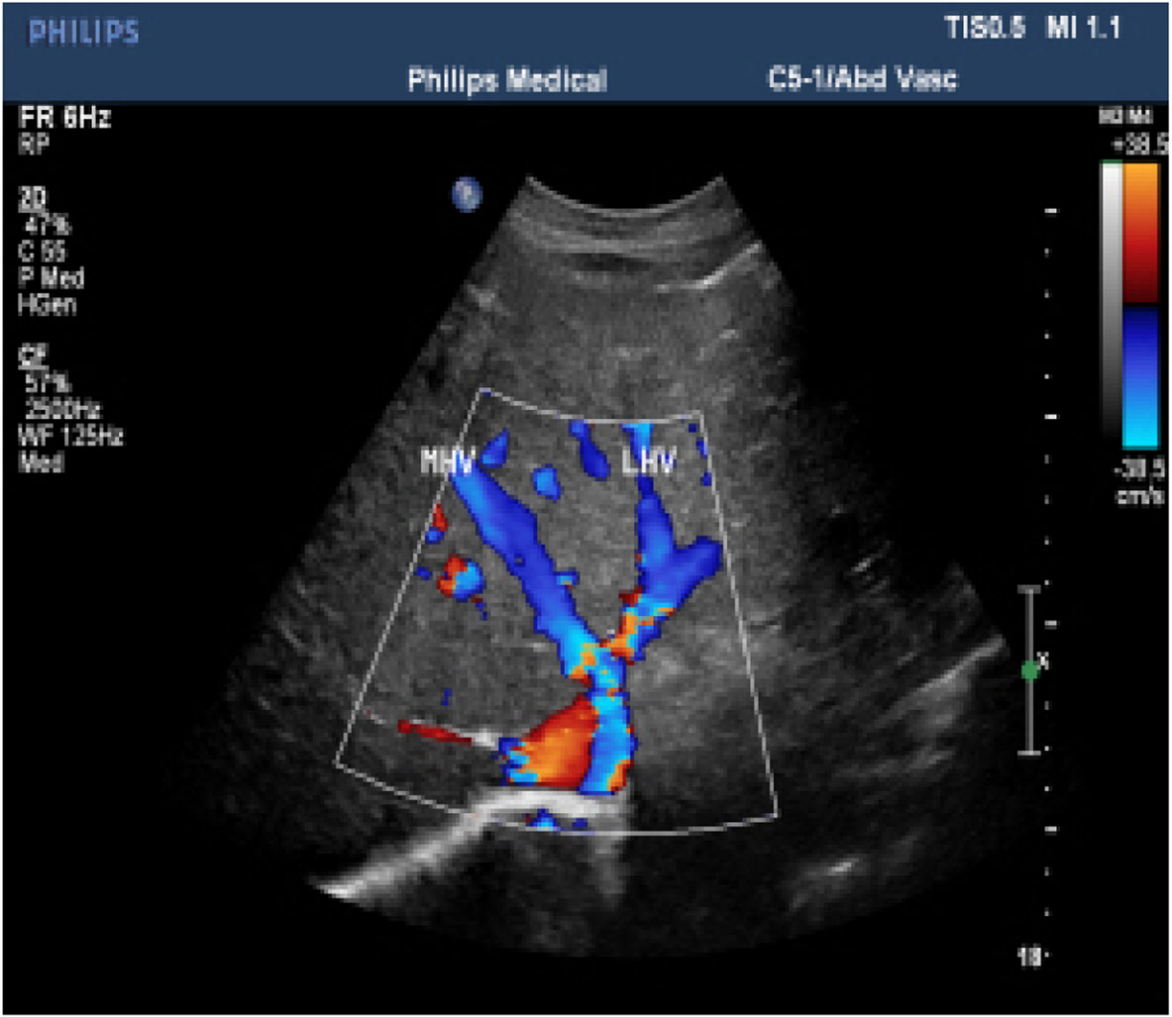
B-ultrasound of the abdomen: Ultrasound shows the left hepatic vein (blood-flow signal is narrow and disordered) and right hepatic vein (blood-flow signal was narrow) are narrow, and the middle hepatic vein is occluded (blood-flow signal is interrupted in the proximal vena cava; the distal end is dilated)

B-ultrasound of the abdomen revealed the caudate lobe to be enlarged (11.6 × 6.0 cm), the left hepatic vein and right hepatic vein to be narrow, and the middle hepatic vein to be occluded (Figs. 2, 3). This imaging modality also revealed liver cirrhosis, splenomegaly (thickness, 6.3 cm; longest diameter, 14.8 cm) and ascites (~ 1.0 cm in front of the liver, ~ 5.7 cm in the abdominal cavity).

**Fig. 4:**
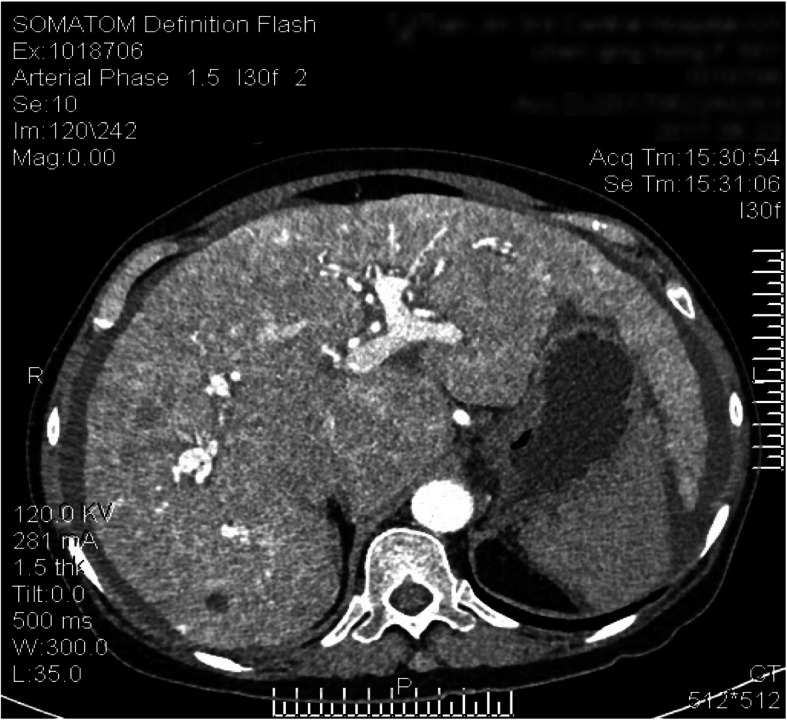
Contrast-enhanced spiral CT of the upper abdomen: This contrast-enhanced CT image shows the portal vein, enhanced liver parenchyma in the arterial phase, and multiple arteriovenous fistulae

**Fig. 5:**
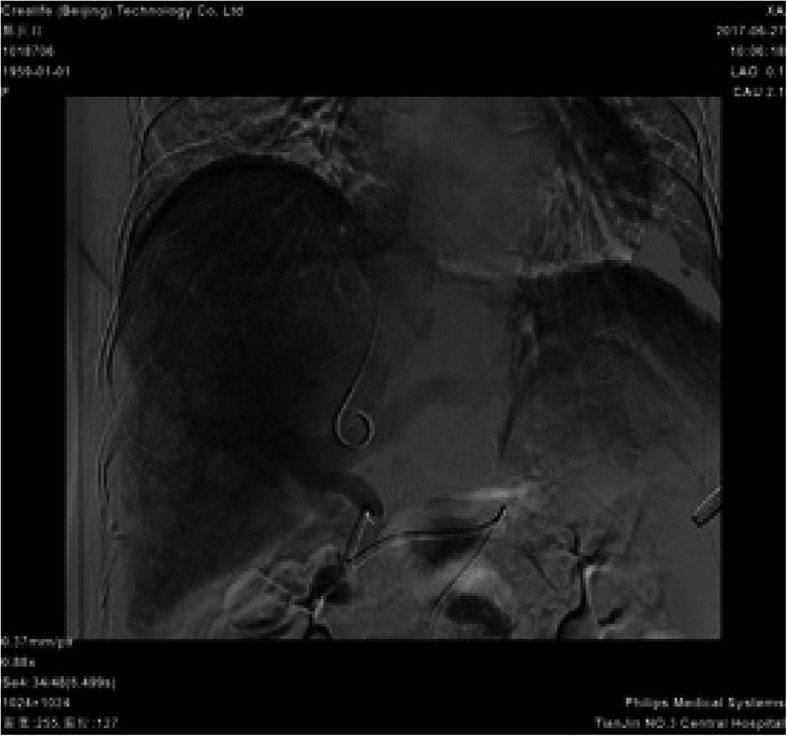
Angiography of liver: Digital subtraction angiography shows the main trunk and branches of the hepatic artery to be tortuous and thickened. The trunk and a branch of the portal vein in the arterial phase can also be seen

**Fig. 6:**
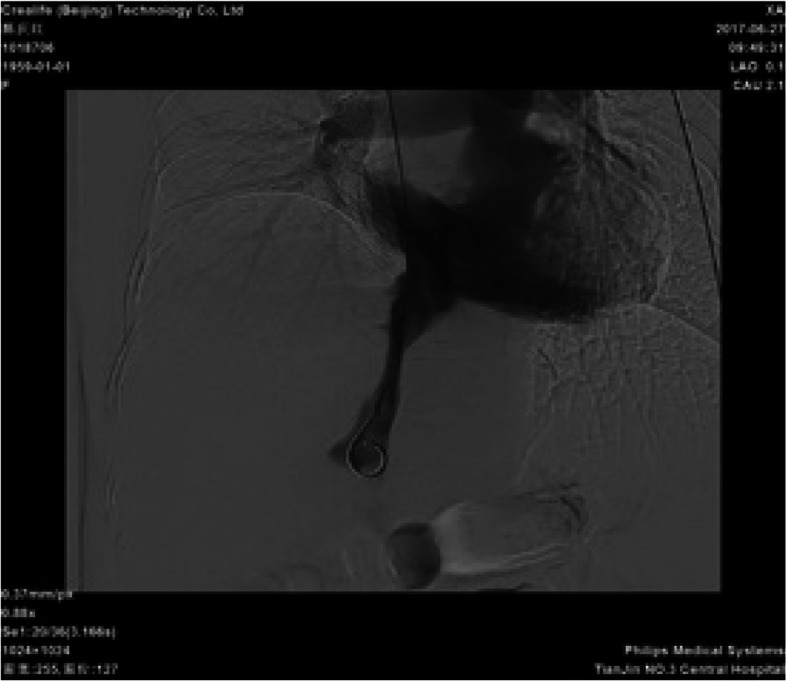
Angiography of liver: Angiography shows a narrow stenosis of the inferior vena cava

**Fig. 7:**
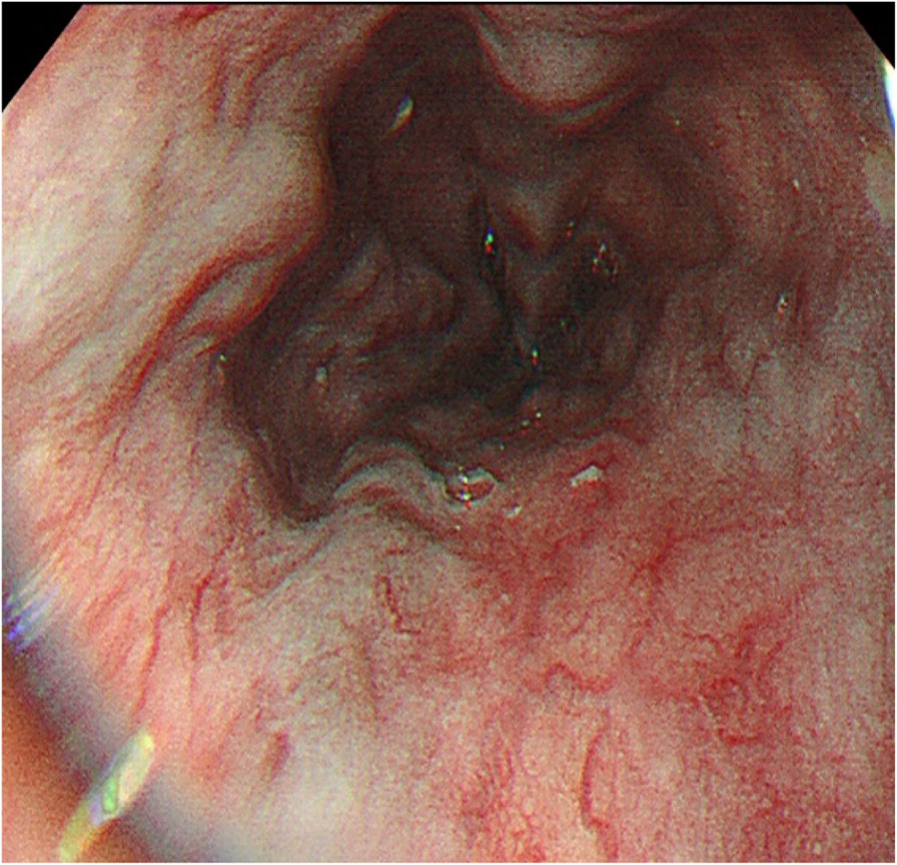
Gastroscopy results: Gastroscopy shows severe esophageal varices

**Fig. 8:**
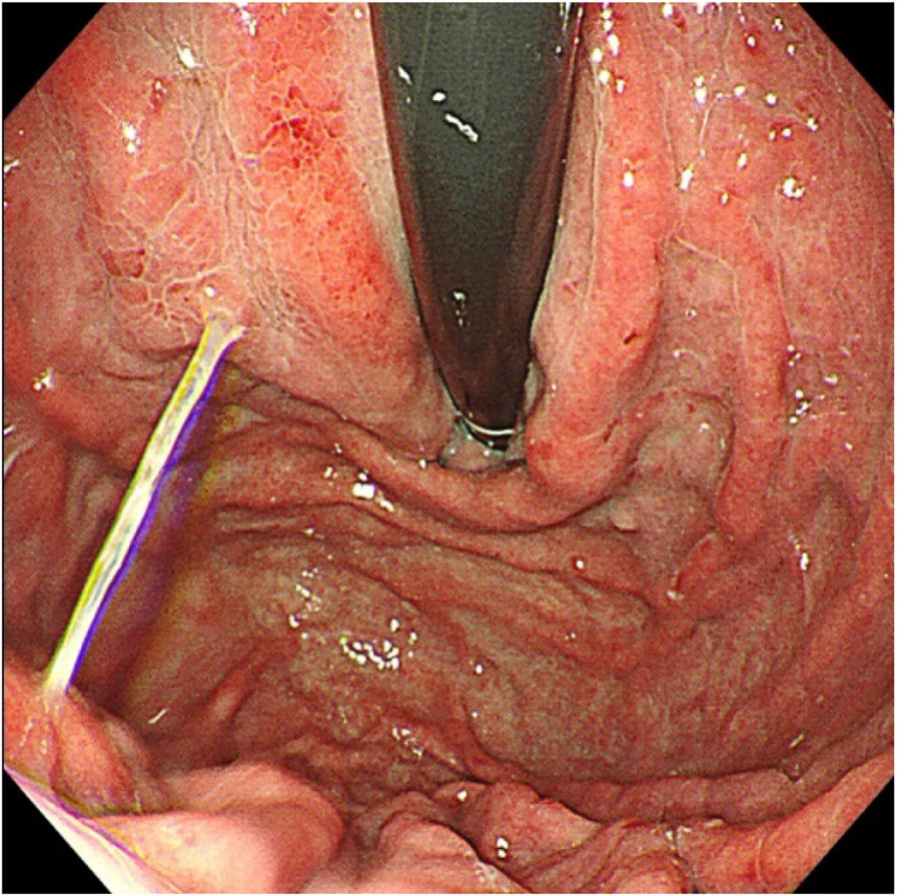
Gastroscopy results: Gastroscopy shows gastric mucosal lesions and mesh-like vascular changes

Contrast-enhanced spiral CT of the upper abdomen showed liver cirrhosis, ascites and formation of multiple arteriovenous fistulae (Fig. 4). Digital subtraction angiography revealed a hepatic arterioportal fistula to be the diffuse type. Angiography showed a narrow stenosis of the inferior vena cava (narrowest diameter, ~ 6 mm) (Figs. 5, 6). Gastroscopy demonstrated esophageal varices and portal hypertensive gastric mucosal lesions (Figs. 7, 8). The hepatic arterioportal fistula and slender hepatic vein meant that occlusion and dilatation, respectively, were unsuccessful.

The patient was diagnosed with cirrhosis, HHT and BCS. We treated her with albumin, diuretics, intermittent peritoneocentesis and subsequent TIPS 1 month later. However, within 2.5 years after discharge from hospital, she still suffered increased ascites, primary peritonitis and epistaxis despite.

Unfortunately, because of the rapid deterioration of the patient’s condition (soon developed intractable ascites, severe hypersplenism and upper gastrointestinal hematorrhea), she didn’t perform the genetic tests and liver biopsy and died on January 2, 2020.

## Discussion and conclusions

HHT is an autosomal-dominant disorder. Type-1 HHT is associated with mutations in the ENG gene that encodes endoglin [2]. Type-2 HHT is associated with mutations in the ACRLV1 gene that encodes activin receptor-like kinase-1 [3]. The lesions can affect the skin and mucous membranes of the nose, mouth, lips, tongue, limbs and internal organs (brain, gastrointestinal tract, liver) [4]. Hepatic involvement is much more common in type-2 HHT [5]. Unfortunately, the patient’s genetic testing and pathology were untouchable. According to her clinical characteristics and family history, the patient was considered to be type-2 HHT.

The pathologic changes wrought by HHT are that the capillaries, arterioles and venules of the affected area become thin. The vessel wall cannot respond to regulation of vascular wall-active substances, and lacks normal systolic and diastolic function [6].

HHHT is defined as all types of hepatic vascular malformations caused by HHT. The microvascular changes in the liver include sinus dilatation and parenchymal fistulae. The early clinical symptoms of HHHT are not obvious. Ianora and colleagues reported only 8.6% of HHHT patients with liver involvement to be symptomatic [7]. With disease development, the decompensation caused by cirrhosis leads to increased levels of transaminase, ascites, as well as esophageal and gastric varices.

According to the criteria revealed by Curacao and colleagues [8], our patient was diagnosed with HHT. DSA (the “gold standard” of BCS diagnosis [9] revealed her to have BCS. HHHT, cirrhosis and BCS coexisted in this patient. It is common to report on HHT complicated with hepatic arterioportal fistula-induced liver cirrhosis, but whether HHHT can lead to BCS has not been studied. We postulate that our patient with BCS had secondary changes in HHHT for two main reasons. First, HHT is characterized by abnormal development of the walls of capillaries, arteries and veins in various parts of the body. The theory of congenital vascular dysplasia is also associated with BCS [1]. BCS is also the least common manifestation of Splanchnic vein thrombosis. HHT is associated with hepatic-vein dysplasia, thin vascular walls, and the vessels cannot relax and contract appropriately. Hence, due to compressed hepatic veins, stenosis and atrophy will hamper the growth and development of the liver. Second, despite an overwhelming propensity for bleeding, HHT patients can also suffer from thrombotic complications [10]. Scholars have reported increased FVIII levels in the general HHT population and that 6–7% of HHT patients have pathologic thromboemboli [11]. Formation of intrahepatic arteriovenous fistulae can lead to hemodynamic changes, resulting in reduced blood flow from hepatic sinuses to the hepatic veins, and so ischemia and hypoxia occur in the hepatic veins. As the disease progresses, the hepatic veins become obstructed due to thrombi (and may even close).

Anticoagulants and/or antiplatelet agents are also recommended to prevent major ischemic or thromboembolic sequelae for HHT patients [10, 12]. In addition, HHHT combined with cirrhosis aggravates ischemia and hypoxia in the hepatic veins further, but the specific mechanism is not clear and further research is needed.

This patient advanced to portal hypertension with refractory ascites, which did not improve significantly after TIPS. We speculate that the reason is related to the long-term portal hypertension caused both by HHHT and BCS.

Liver transplantation (LT) may be considered to be the only cure for HHT [13–15] and hepatic arterioportal fistula [16], although the role and curative action of LT in patients with HHT is controversial [17].

We report a case of progressive cirrhosis with HHT and BCS. In our case, BCS may be secondary to HHT. Further studies are required to evaluate the relationship between HHHT and BCS but our observations already challenge the TIPS therapeutic strategy in BCS secondary to HHHT patients. In addition, it could result in a better understanding of the mechanisms of liver involvement by HHT.

## Data Availability

The datasets used and/or analyzed during the current study are available. from the corresponding author on reasonable request.

## Abbreviations

TIPS: Transjugular intrahepatic portosystemic shunting
HHT: Hereditary Hemorrhagic Telangiectasia
HHHT: Hepatic Hereditary Hemorrhagic Telangiectasia
BCS: Budd–Chiari syndrome
CT: Computed tomography
LT: Liver transplantation
